# Biology, Genetic Diversity, and Conservation of Wild Bees in Tree Fruit Orchards

**DOI:** 10.3390/biology12010031

**Published:** 2022-12-24

**Authors:** Olivia Kline, Ngoc T. Phan, Mitzy F. Porras, Joshua Chavana, Coleman Z. Little, Lilia Stemet, Roshani S. Acharya, David J. Biddinger, Gadi V. P. Reddy, Edwin G. Rajotte, Neelendra K. Joshi

**Affiliations:** 1 Department of Entomology and Plant Pathology, University of Arkansas, Fayetteville, AR 72701, USA; 2 Research Center for Tropical Bees and Beekeeping, Vietnam National University of Agriculture, Gia Lam, Hanoi 100000, Vietnam; 3 Department of Entomology, Pennsylvania State University, University Park, PA 16802, USA; 4 Department of Biology, University of Central Arkansas, Conway, AR 72035, USA; 5 Penn State Fruit Research and Extension Center, Biglerville, PA 17307, USA; 6 USDA-ARS-Southern Insect Management Research Unite, 141 Experiment Station Rd., P.O. Box 346, Stoneville, MS 38776, USA

**Keywords:** Megachilidae, orchard bees, mason bees, pollinators, pesticides, genetic diversity, mining bees, conservation, wild bees

## Abstract

**Simple Summary:**

Honey bees (*Apis mellifera*) are the most economically important agricultural pollinator in North America, as well as being the most frequently studied bee species. Many agricultural systems, such as fruit tree orchards, benefit from having a diversity of bee species present. In this article, we present information about the types of bees that can be found in orchards and explore their mating behaviors, life cycles, genetic differences, flower preferences, and foraging activities. Many orchard-pollinating bees, including bumble bees (*Bombus* spp.), mason bees (*Osmia* spp.), and mining bees (*Andrena* spp.), are often less studied than honey bees. All bees encounter threats to their health and behavior while out foraging. The impacts and mitigation of these threats are often better understood in honey bees. This review summarizes the current knowledge of these threats to orchard bees’ health, identifies gaps in the knowledge, and discusses potential management and conservation practices.

**Abstract:**

Different species of bees provide essential ecosystem services by pollinating various agricultural crops, including tree fruits. Many fruits and nuts depend on insect pollination, primarily by wild and managed bees. In different geographical regions where orchard crops are grown, fruit growers rely on wild bees in the farmscape and use orchard bees as alternative pollinators. Orchard crops such as apples, pears, plums, apricots, etc., are mass-flowering crops and attract many different bee species during their bloom period. Many bee species found in orchards emerge from overwintering as the fruit trees start flowering in spring, and the active duration of these bees aligns very closely with the blooming time of fruit trees. In addition, most of the bees in orchards are short-range foragers and tend to stay close to the fruit crops. However, the importance of orchard bee communities is not well understood, and many challenges in maintaining their populations remain. This comprehensive review paper summarizes the different types of bees commonly found in tree fruit orchards in the fruit-growing regions of the United States, their bio-ecology, and genetic diversity. Additionally, recommendations for the management of orchard bees, different strategies for protecting them from multiple stressors, and providing suitable on-farm nesting and floral resource habitats for propagation and conservation are discussed.

## 1. Introduction

Many commercially grown orchard crops, including apples, pears, almonds, cherries, oranges, and cashews, provide vital nutrients to the human diet. For example, tree nuts, such as Brazil nuts, almonds, and cashews, are high in lipid and protein contents [1]. Citrus and other fruits, including apples and cherries, are the primary sources of vitamin C in many areas of the world, not just due to their moderate to high vitamin C content, but also because of their popularity [2]. These crops can benefit significantly from insect pollination, particularly from bees, resulting in higher yields and better-quality fruit when bee pollinators are present [1,3,4,5]. Orchard-pollinating bees, therefore, play an important role in improving the diet of many consumers and increasing profits for fruit and tree nut producers [6,7,8].

Honey bees (*Apis mellifera*) are the best-known bee species and the most widely used in many cropping systems, due to their large workforce, ease of transport, and long history of commercial use [9]. There are, however, many other bee species, both wild and commercially managed, which can be found in orchards and contribute to orchard pollination. Many native North American bees, such as mason bees, mining bees, cellophane bees, sweat bees, bumble bees, and carpenter bees, can all be found in orchards [10,11,12,13,14,15,16]. Despite this wide diversity of bees in orchard cropping systems, most research into bee biology, population declines, and mitigation measurements have focused on honey bees.

The population size and range of many North American bee species have declined recently, as has been well documented in several native bumble bees (*Bombus* spp.) [17,18,19]. However, such information on the abundance and population trends for solitary species is limited to a few studies [20]. Numerous studies have shown that multiple stressors, including pathogens and parasites, insufficient food sources, habitat disturbance, pesticide exposure, and climate change may contribute to these bee declines and, with them, declines in the yield of the crops they pollinate [21,22,23,24].

Understanding the ecology and distribution of bee species, in addition to the increasing awareness of pollinator health issues, habitat, and management, are crucial steps in protecting and supporting bee populations [25,26,27]. This comprehensive review will cover current knowledge of the bioecology, phylogeny, and management of orchard-pollinating bees and highlight mitigation strategies to help protect wild and managed bee populations.

## 2. Types of Bees Found in Orchards

Although honey bees are the best-known bee species globally, many other species, including many North American native bees, can be found pollinating orchard crops. These non-*Apis* bees can be more efficient pollinators for certain orchard crops than honey bees [28,29]. Most of these indigenous bees are wild bees and are often known as alternative pollinators [30]. Additionally, having a diversity of bee species in an area can improve the quality and yield of many crops [16,31].

**Figure 1:**
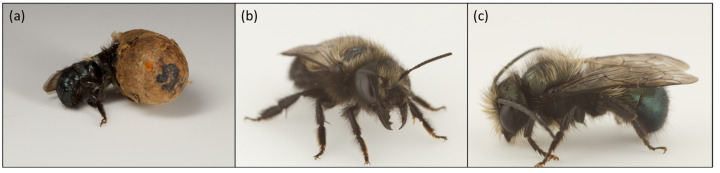
A blue orchard bee, *Osmia lignaria*, emerging from an overwintering cocoon (**a**), and female (**b**) and male (**c**) bees. Pictures by C.Z. Little.

One common group in orchards are mason bees (*Osmia* spp.), tunnel-nesting solitary bees that use mud in their nest construction [32,33]. There are approximately 500 known *Osmia* species globally, 140 of which are native to the United States and Canada [32,34]. They emerge in the spring and early summer when the weather is about 10–15 °C. Mason bees are generalist feeders and forage on a wide variety of flowering plants. Their short flight range and preference for nectar and pollen from fruit trees make them efficient and capable orchard pollinators, because they tend to stay within orchard blocks [35,36,37]. They are low maintenance to keep, requiring only a nesting box to be provided in the springtime and then placed in an insectary under ambient temperature over the winter [32,34,35,36]. One species, *Osmia cornifrons* (Hymenoptera: Megachilidae), has been commercially managed to pollinate apples in Japan since the 1930s, and was introduced in the United States in 1970s for the same purpose. Other *Osmia* species have a history of commercial use, as well, such as *O. lignaria* (Figure 1) for almond pollination in the United States and *O. bicornis* for pears in Europe [33,38,39,40,41,42].

Mining bees (*Andrena* spp.) can also contribute to orchard pollination. They are solitary bees that build nests in underground tunnels [43,44,45]. They can be frequently found in temperate regions with open, usually sandy or dry soil, nesting habitats. In the United States, their range extends from Texas to Florida and up to the East Coast. There are approximately 1,400 species around the world, and about 400 native bee species in North America [14,46]. The foraging preferences of mining bees vary depending on the species, but most are specialists, making them strongly associated with certain flower species [47,48,49,50,51]. Mining bees are primarily active in the spring, and they are important pollinators of many early blooming crops, including blueberries and apples [49,52,53].

Cellophane bees, also called polyester bees (*Colletes* spp.), are solitary ground-nesting bees that line their nest with a waterproof polyester substance [54,55]. Cellophane bees are often generalist feeders and forage on various trees and shrubs which blossom in the spring, such as maples, chokecherries, gooseberries, and blueberries. However, some specialize, such as *C. validus* on blueberries. Approximately 460 species are known to exist worldwide, with about 100 species in North America [14]. In the United States, their geographic distribution ranges from New Hampshire to Michigan through the Mid-Atlantic states [56,57,58].

Sweat bees (Hymenoptera: Halictidae) are small bees that can be attracted to the salt in human perspiration. Similar to other non-*Apis* bees, they feed on flower pollen and nectar, but also need to supplement their diet with moisture and salts [59]. Most sweat bees are solitary, although there are several eusocial species, such as *Lasioglossum zephyrus*, *L. imitatum*, *L. tegulare*, *L. pilosum*, *L. cressonii*, and *L. vierecki* [60]. Most species nest in burrows in the ground, but some prefer to nest in rotting wood [59,61]. 

Similar to honey bees, bumble bees (*Bombus* spp.) are a social group and can be highly successful orchard pollinators. They are referred to as “bumble” bees due to their ability for sonication, or “buzz pollination,” in which they vibrate their flight muscles to encourage pollen release from certain type of flowers [29]. There are over 250 species of bumble bees in the world, with 54 species in the United States [62]. Commercially, they are often used for greenhouse pollination services, although they pollinate many other flowers, including blueberries, cranberries, strawberries, and kiwis [60,61,62,63]. They are especially effective in cold weather because they can thermoregulate. However, their colonies are smaller in the spring, compared to honey bees, so there are fewer bumble bee workers out foraging. 

Large carpenter bees (*Xylocopa* spp.) are also valuable pollinators capable of sonication, but are often considered pests because they bore into wood to construct their nests [54]. They are solitary bees that emerge in early spring after overwintering in old nest tunnels. They can resemble bumble bees, but typically have shinier and less hairy abdomens. In some crops, such as blueberries, carpenter bees ‘steal’ nectar by piercing the flower corolla, bypassing the flower’s reproductive apparatus. However, this nectar robbing behavior still provided pollination and did not negatively affect fruit set of the blueberries [63].

### 2.1. Based on Nesting Behavior

Bees found in fruit tree orchards can be grouped into three distinct nesting types: ground-nesting, tunnel-nesting, and cavity-nesting (Table 1). Of all the known solitary bees, 70% are ground-nesting [61]. A few species utilize wood and pith for nesting (Table 1). An example of ground-nesting bees located in orchards are the *Augochlora* species (Hymenoptera: Halictidae). Although most sweat bees nest in well-drained soil, these *Augochlora* spp. bees mainly nest in wood. To select a suitable nest, the female will look for wood that remains moist throughout the warm summer months [61]. Suitable nests are often found in wooded valleys, next to streams, or on the northside face of a hill [61]. Once suitable wood is found, the bee will excavate a cavity with a cluster of cells supported by pillars or create the cells adjacent to the wood walls linearly [64]. Each cell contains a single egg, male or female, and a provision of pollen and nectar for the offspring. The pillars and cell partitions are used for support and are constructed from the phloem and xylem of trees; the female may use her Dufour’s gland to convert these substances into a waxy material which can be manipulated for nest construction [65]. This role of the Dufour’s gland is speculative, however, and remains primarily unexplored [66].

Many of the bees found in orchards are tunnel-nesting bees. These bees typically locate pre-established tunnels such as hollow plant stems, abandoned insect burrows, and snail shells [67]. Mason bees (*Osmia* spp.) are well-known tunnel-nesting bees. They typically select a nest based on several criteria: the proximity to other nesting bees, previous use of the nest, proximity to a food source, and the length and diameter of the potential nesting cavity [68,69]. Once a female selects a nesting site, she gathers materials such as mud, pebbles, and leaves to construct cell partitions. Different species collect different materials. For example, *Osmia lignaria propinqua* solely uses wet mud to create mud partitions, whereas *Osmia montana montana* only collects leaves to masticate into pulp and use as cell partition material and *Osmia californica* cuts pieces of leaves, rolls them in soil, and uses the pulp–soil mixture for cell partitions [70]. The bee then begins by creating the back wall of the first cell deepest in the cavity. She leaves a provision of pollen and nectar, lays an egg, and seals the cell with a mud partition [71]. The female will continue to repeat this pattern until the nest is complete and the final partition is a mud plug to protect the nest [72]. For mason bees, the deepest cells contain female eggs, while the outermost cells contain male eggs [68].

**Table 1:** Common bee species found in orchards. Each species is listed with their known nesting behavior, foraging specialization, and their social behavior *.

Species	Nesting Behavior	Foraging Specialization	Social Behavior
*Agapostemon virescens*	Ground	Generalist	Solitary
*Andrena barbara*	Ground	Generalist	Solitary
*Andrena barbilabris*	Ground	Generalist	Solitary
*Andrena bisalicis*	Ground	Generalist	Solitary
*Andrena bradleyi*	Ground	Vaccinium specialist	Solitary
*Andrena carlini*	Ground	Generalist	Solitary
*Andrena carolina*	Ground	Vaccinium specialist	Solitary
*Andrena cineraria*	Ground	Generalist	Solitary
*Andrena commoda*	Ground	Generalist	Solitary
*Andrena crataegi*	Ground	Generalist	Solitary
*Andrena cressonii*	Ground	Generalist	Solitary
*Andrena dunning*	Ground	Generalist	Solitary
*Andrena forbesii*	Ground	Generalist	Solitary
*Andrena hippotes*	Ground	Generalist	Solitary
*Andrena imitatrix*	Ground	Generalist	Solitary
*Andrena mandibularis*	Ground	Generalist	Solitary
*Andrena miserabilis*	Ground	Generalist	Solitary
*Andrena nasonii*	Ground	Generalist	Solitary
*Andrena nuda*	Ground	Generalist	Solitary
*Andrena perplexa*	Ground	Generalist	Solitary
*Andrena pruni*	Ground	Generalist	Solitary
*Andrena rugosa*	Ground	Generalist	Solitary
*Andrena tridens*	Ground	Generalist	Solitary
*Andrena vicina*	Ground	Generalist	Solitary
*Andrena violae*	Ground	Viola specialist	Solitary
*Andrena wilkella*	Ground	Generalist	Solitary
*Anthophora abrupta*	Ground	Generalist	Solitary
*Augochlora pura*	Wood	Generalist	Solitary
*Augochloropsis metallica*	Ground	Generalist	Primitively Eusocial
*Bombus balteatus*	Cavity	Generalist	Eusocial
*Bombus bifarius*	Cavity	Generalist	Eusocial
*Bombus bimaculatus*	Cavity	Generalist	Eusocial
*Bombus flavifrons*	Cavity	Generalist	Eusocial
*Bombus griseocollis*	Cavity	Generalist	Eusocial
*Bombus impatiens*	Cavity	Generalist	Eusocial
*Bombus perplexa*	Cavity	Generalist	Eusocial
*Bombus sylvicola*	Cavity	Generalist	Eusocial
*Bombus terrestris*	Cavity	Generalist	Eusocial
*Bombus vagans*	Cavity	Generalist	Eusocial
*Ceratina calcarata*	Pith	Generalist	Sub-social
*Ceratina dupla*	Pith	Generalist	Sub-social
*Ceratina strenua*	Pith	Generalist	Sub-social
*Colletes validus*	Ground	Vaccinium specialist	Solitary
*Habropoda laboriosa*	Ground	Vaccinium specialist	Solitary
*Halictus confusus*	Ground	Generalist	Primitively Eusocial
*Halictus ligatus*	Ground	Generalist	Primitively Eusocial
*Halictus rubicundus*	Ground	Generalist	Sub-social
*Hoplitis adunca*	Tunnel	Boraginaceae specialist	Solitary
*Hylaeus punctulatissimus*	Ground	Lamiaceae specialist	Solitary
*Lasioglossum admirandum*	Ground	Generalist	Primitively Eusocial
*Lasioglossum cressonii*	Wood	Generalist	Primitively Eusocial
*Lasioglossum foxii*	Ground	Generalist	Solitary
*Lasioglossum imitatum*	Ground	Generalist	Primitively Eusocial
*Lasioglossum pilosum*	Ground	Generalist	Primitively Eusocial
*Lasioglossum quebecense*	Ground	Generalist	Solitary
*Lasioglossum tegulare*	Ground	Generalist	Primitively Eusocial
*Lasioglossum truncatum*	Ground	Generalist	Primitively Eusocial
*Lasioglossum versans*	Ground	Generalist	Primitively Eusocial
*Lasioglossum versatum*	Ground	Generalist	Primitively Eusocial
*Lasioglossum vierecki*	Ground	Generalist	Primitively Eusocial
*Lasioglossum zephyrus*	Ground	Generalist	Primitively Eusocial
*Megachile addenda*	Ground	Fabaceae specialist	Solitary
*Melitta americana*	Ground	Vaccinium specialist	Solitary
*Osmia apicata*	Tunnel	Onosma specialist	Solitary
*Osmia atriventris*	Tunnel	Generalist	Solitary
*Osmia bicornis*	Tunnel	Rosaceae specialist	Solitary
*Osmia californica*	Tunnel	Generalist	Solitary
*Osmia cerinthidis*	Tunnel	Boraginaceae specialist	Solitary
*Osmia cornifrons*	Tunnel	Generalist	Solitary
*Osmia cornuta*	Tunnel	Generalist	Solitary
*Osmia lignaria*	Tunnel	Generalist	Solitary
*Osmia maxillaris*	Tunnel	Fabaceae specialist	Solitary
*Osmia maxschwarzi*	Tunnel	Fabaceae specialist	Solitary
*Osmia montana*	Tunnel	Generalist	Solitary
*Osmia pumila*	Tunnel	Generalist	Solitary
*Osmia scheherazade*	Tunnel	Fabaceae specialist	Solitary
*Osmia taurus*	Tunnel	Generalist	Solitary
*Osmia virga*	Tunnel	Vaccinium specialist	Solitary
*Peponapis pruinosa*	Ground	Cucurbita specialist	Solitary
*Xylocopa virginica*	Wood	Generalist	Semi-social

* Some information is based on Wolf and Ascher [73], Lerman and Milam [74], Wood and Roberts [75], Dar et. al 2021 [76], Scott et al. 2016 [77], Weaver and Mallinger 2022 [78], Zurbuchen et al. 2010 [79], Fowler 2016 [80], Haider et al. 2014 [81], and Muller 2012 [82].

Cavity-nesting bees can also be commonly found in orchards. The most well-known cavity-nesting bees are eusocial honey bees (*Apis* spp.). These bees can grow in large colonies of up to 50,000 individuals [83]. When the colony has a newly mated queen bee, the bees will swarm—many of the bees will leave with the old queen, while the others stay with the new queen [84]. Hundreds of scout bees will leave the swarm to select a new home, explore, and communicate their findings through a waggle dance [84]. The bees will select a nest based on the height of the cavity, the cavity entrance, the volume of the cavity, and the distance from the old nest [85,86]. Such cavities can be found in trees, rock crevices, and man-made structures. Once a new nest is selected, the entire swarm will migrate to their new home. Worker bees will begin to synthesize wax to line the cavity and create hexagonal-shaped combs [86]. Similar to ground-nesting and tunnel-nesting bees, the queen honey bee lays only one egg per cell, with male drones toward the outside of the nest and females toward the middle [85]. Only one part of the hive, the brood chamber, is allocated to progeny rather than the entire nest, as other parts of the hive are used for honey and pollen storage [85]. Additionally, the queen bee lays female eggs throughout most of the year and only lays male eggs when the mating season nears [87].

Bumble bees (*Bombus* spp.) may grow into large colonies of up to 1,700 individuals [88]. Unlike *Apis* spp., where both the queens and workers overwinter, bumble bee queens mate and then overwinter alone in the soil, while the rest of the colony dies out [89]. The following spring, the bumble bee queen will emerge and search for a new nest. Nesting sites are usually selected based on the drainage level, heat absorption, and shelter degree, as well as other species-specific needs [90]. Once a nesting site is chosen, the queen begins collecting pollen and nectar, and synthesizing wax to construct honey pots. The honey pots allow the queen to store nectar while she lays eggs and cares for the brood. As with honey bees, the bumble bee queen lays female eggs most of the year, while males are only produced near mating season in late summer or fall [91].

### 2.2. Based on Foraging Preferences

Host plant selectivity in bees is classified into two categories: generalist feeders, which collect pollen from a wide range of plant families, and specialists, which specialize in a single species, genus, or family of flowering plants. The pollination services provided by these two groups vary in the efficiency and diversity of the pollination service provided.

Bee species vary greatly in their foraging behaviors and floral preferences. For instance, male bees and parasitic bees do not collect pollen, though they still visit flowers for nectar. Bees that visit flowers solely for nectar can still contribute to pollination, however, as pollen can still be transferred by the bee’s external setae. The difference in pollination service between bees visiting flowers for pollen and nectar, versus bees visiting for nectar alone, is poorly studied [92,93]. There is evidence that most pollen transfer occurs via these external hairs, because pollen carried in scopa is often moistened, thus decreasing the chance for pollen transfer [94]. Flower visits result in pollen transfer, as seen in flies, wasps, and beetles visiting flowers for nectar and not collecting pollen [93]. For bees, this means that all flower visits have the potential for pollen transfer, but active pollination is more efficient [95]. To build on this complexity, bee species have different structures of hairs, in varying densities, on different parts of their bodies. Megachilid bees carry pollen on scopae formed on the underside of their abdomen, whereas other bees have scopae on their hind-femora or hind-tibiae [92]. The thickness and body position of bee scopae and external body hairs, as well as the shape and pollen structures of flowers can all impact the efficiency of pollen transfer [95]. Additionally, some bees, such as *Hylaeus* spp., do not collect pollen externally, but rather internally in their crop, which can reduce the amount of pollen transferred to the flowers they visit [96].

Generalist and specialist tendencies can also vary throughout the year. Different bee species have varying times of the year when they are active and visiting flowers. Some are only active for one month out of the year, such as *Osmia lignaria*, whereas others, such as *Apis mellifera*, can be active year round, as long as the weather is warm enough [97]. A bee species’ breadth of floral fidelity thus depends on the composition of flowers present at a given time [98]. Bees active for a few months or more will typically have greater options of floral resources from which to forage, whereas those with smaller active seasons typically have access to fewer floral resources. Orchard species tend to bloom earlier in the year than many other flowering plant species, which makes them an important and attractive food source for many early season pollinators, especially those with short activity periods [99].

When there is a large breadth of floral resources available, generalist species are documented visiting a wide range of plant species, though individuals within the species may visit a specific set of plant species. This varies at different times of the year or when different plant species are blooming. For example, the common eastern bumble bee (*Bombus impatiens*) tends to have some individuals specializing and others generalizing in their pollen foraging [100,101]. A study by Kratochwil et al. (2009) documented the floral visiting range of some generalist bees to be between 2 and 22 different plant species [102]. Therefore, a bee species may be classified as a generalist, but that term refers to the species’ tendencies as a whole and may not reflect the foraging behavior of a specific colony or bee.

Generalist bees provide pollination to a wide array of plant species, but can still have distinct floral preferences. *Osmia lignaria*, for example, can visit and feed from a variety of plants, but has shown a preference for fruit trees, with their nests containing 85% to 100% of pollen originating from orchard tree species [99]. Similarly, *Andrena* spp. have high proportions of both apple and blueberry pollen within their nests [103]. For instance, *A. barbilabris*, *A. carlini*, *A. crataegi*, *A. vicina*, *Agapostemon virescens*, *C. calcarata*, *P. pruinosa*, *H. ligatus*, *L. tegulare*, and *O. atriventris* were documented carrying pollen from apple, blueberry, and other non-orchard species [103]. In a study by Scott et al. (2016), *Andrena carlini* and *A. vicina* were observed as having 51% and 69% blueberry pollen loads, respectively [77]. *Anthophora abrupta* is a soil-nesting generalist with a wide range of plant species it will pollinate, as well as crop such as species cranberry, blackberry, raspberry, and tomato [104]. Other important generalist orchard bees including *Bombus* and *Xylocopa* spp. can have broader foraging preferences. These broad generalist feeders can be highly important in orchards, as they provide pollination services to crops and support the diversity of other flowers. This can help provide floral resources and bolster the entire bee assemblage in orchards. The number of generalist bee species that have been documented visiting orchard flowers is large and growing, but also indicates that the enhancement of bee communities in general is good for orchard crop production. By providing redundant pollinators, the requirements of orchard pollination can be met even if some species are facing decreases in abundance.

Specialist bees tend to feed within a certain genus of plants, as within the genus, flower structures are often similar [92]. There are many specialist behaviors that have been documented, with some bees visiting a single species, and others feeding on several species or genera within a family of plants. *Osmia* spp. tend to be generalists, although some species, such as *O. apicata*, *O. cerinthidis*, *O. maxillaris*, *O. maxschwarzi*, and *O. scheherazade*, have been documented as only visiting flowers from a single family of plants [81]. There has been some concern that climate change could disrupt the synchrony of obligate species phenology, but the few studies investigating this show that any shift in one species phenology is present in other species as well [105].

For orchards, enhancing specialist pollination population would likely focus on bees that specialize in pollinating the orchard crop, though the presence of specialists for non-crop plants could also indicate healthy pollinator assemblages. *Habropoda labriosa*, *A. bradleyi*, *A. carolina*, *Melitta americana*, *M. addenda*, *C. validus*, and *O. virga* are *Vaccinium* spp. specialists, making them important for yields in blueberries [106,107]. For exotic Rosaceae orchard species such as apple, pear, cherry, and peach, there are not many native species documented that specialize in their pollination. However, many *Andrena*, *Osmia*, and *Colletes* spp. are active in early spring and have a strong preference for the pollen and nectar of those orchard species [108]. Orchard specialists tend to be less common, but for crops with identified specialists, their presence could increase pollination efficacy.

## 3. Bioecology of Major Species

### 3.1. Mating Behavior

Many orchard-pollinating bees are sexually dimorphic, with the males having a smaller body size, longer and more segmented antennae, lighter facial hair coloration, and no stinger [72]. Adult males will feed on nectar but not gather pollen; thus, they also lack the pollen-collecting structures, such as scopae or corbiculae, which are present on female bees. In the case of social bees, such as *Apis* and *Bombus* spp., there is an additional caste differentiation among females. Queen bees are larger and have fully developed ovaries, whereas workers are smaller and usually do not produce offspring [107].

Tunnel-nesting and ground-nesting orchard bees are often protandrous, meaning that the males emerge first from the nests and begin to patrol nesting sites for females and nectar [68,72]. Females emerge later, coinciding with orchard blooms in the spring [71]. Once females emerge, they release a sex pheromone that attracts nearby males [108]. A male bee then mounts the female and embraces it with his hind legs. In this position, the male bee vibrates his thorax, producing a buzzing sound while simultaneously rubbing his antennae with his forelegs [108]. At this time, the female bee uses both the thoracic vibrations and the released chemicals to select a suitable partner [109]. Once mated, females become less attractive and more unreceptive, making them monandrous. However, some females in bee species have been shown to mate more than once [72,110].

Bumble bees and honey bees differ because males emerge from a brooding chamber in the hive rather than a nest; males still congregate before females arrive. Unlike other bee species, queen honey bees purposefully mate with multiple drones in midair at drone congregation areas [83]. Honey bee drones die after mating because their copulatory and other organs are ripped out and left with the queen bee [111].

### 3.2. Life Cycle

**Figure 2:**
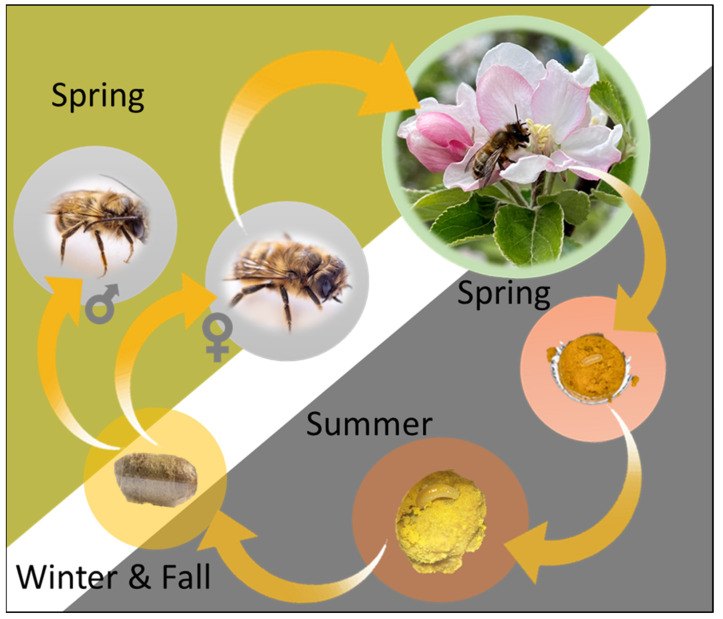
Life cycle of a tunnel-nesting bee: *Osmia corniforns*, which is dependent on spring-flowering fruit trees. Similar to many other *Osmia* species, after mating, female bees collect pollen from flowers of various fruit trees and store them in nest cells, deposit eggs, and then individually seal the nest cells. Upon hatching, larvae complete development during the summer and pupate while the adult overwinters to emerge next spring. Concept illustration and pictures by N. Phan and N. Joshi.

The life cycle of all bees consists of four stages: egg, larva, pupa, and adult. However, each species can vary in the time of emergence, life span, and nesting structure. Tunnel-nesting mason bees, such as *O. lignaria* (blue orchard bees, Figure 1) [71] and *Osmia cornifrons* (Japanese orchard bee, Figure 2), emerge in early spring. Females choose a suitable mate, fill their spermatheca, and then after 1–2 days, the female begins to locate a nesting site [72]. Once a female selects a suitable site, she begins constructing the nest by collecting mud and other materials. The eggs are laid in a linear series of cells with mud partitions in between each cell. During early summer, the eggs undergo five larval instars. The first instar remains within the egg, feeding on chorion, and then the larva hatches out in the second instar [68]. The fifth instar spins a silk cocoon, although it does not undergo pupation immediately. Instead, it stays as a prepupa in a period of diapause lasting 1–2 months, depending on the temperature and geographical location [69,71]. By late summer, the prepupa molts into a pupa; the pupa develops all adult structures except the wings [68,69]. About one month later, the pupa molts again into a fully grown adult and overwinters inside its pupal cocoon until the next spring, when it emerges and begins the cycle again [69].

Similarly, other tunnel-nesting species emerge in early spring, such as the sweat bee, *Augochlora pura*. Female bees search for an abandoned burrow (tunnel) of some other insect to utilize as a starting point for their nest [112]. Then they use phloem and xylem from trees to construct their nests. Once the eggs are laid, the female seals the nest to protect the brood [61]. The eggs hatch into larvae, which begin to consume the pollen and nectar provision [113]. Prior to pupation, the larvae enters a prepupa resting stage. Once the optimal environmental factors have been achieved, the larvae pupate into adults and leave the nest in late summer or early fall to mate. Mated female bees will overwinter, while the male bees die out for the year [112].

Honey bees overwinter by huddling together in a cluster to generate warmth, while feeding on their honey stores. They become active again during the late winter or early spring [114]. This is characterized by established queen bees resuming their egg-laying activity. Once the eggs are laid, it takes them approximately three days to hatch into larvae [115]. Regardless of their future role, the larvae are fed royal jelly for the first 2–3 days; at the end of the third day, queen bee larvae continue to be fed royal jelly, while drone and worker bee larvae are fed pollen, nectar, and water [116]. After an average of six days, the larvae spin cocoons and enter a prepupal stage. Hive workers seal the pupae in their cells by covering the cells with wax caps [87]. The pupa metamorphosize into an adult. The new adult bee will then chew its way out of the cell and service the hive through its respective role [115]. On average, queen bees take 16 days to develop, workers take 21 days, and drones take 24 days. Drones are only produced in the spring/summer [87].

### 3.3. Foraging Behavior of Orchard Bees

Not much is known about the foraging distance for the thousands of bee species around the world. The complexity and variation among and within species make foraging distance difficult to study and determine. Foraging distances are reported as maximums and averages, and average distances typically have high variability. Foraging distances have been measured in many ways, including mark–recapture, harmonic radar, pollen analysis, feeder training, and homing experiments. These measure different aspects of bee flight, and it is unclear which method is a better indicator of how far bees forage on average [79]. Homing (translocation) experiments measure the maximum distance a bee can fly or, potentially, their ability to relocate their nest [79]. Therefore, homing does not replicate natural flight and is not considered to be an indicator of foraging range. For the other methods, many variables exist when determining foraging distances, and more information is needed to determine how far species will travel to forage in natural systems. Foraging distances vary within species as well, although the majority of individuals stay relatively close to their nests. Data from Zurbuchen et al. (2010) investigated foraging distances of *Hylaeus punctulatissimus* and *Hoplitis adunca* and found that 50% of females were not documented flying farther than 225 m and 300 m, respectively, whereas 75% did not exceed 400 m and 700 m. The maximum distances recorded were 1100 m and 1400 m, respectively [79]. This shows that, although some individual bees may forage at greater distances, most forage at a fraction of the maximum distances recorded.

Some factors can be used to predict the foraging distance for bee species. The average foraging distance an individual will travel from their nest has been correlated with their inter-tegular span (ITS). The ITS is a relative size measurement, so it can be said that larger bees tend to travel a greater distance to gather pollen when compared with smaller bees [117]. However, this size correlation does not predict how far bees will forage, because the quality of foraging resources in an area also impacts the distance they will travel to gather pollen [15]. As the distance from nest to flower, the density of flowers, and the amount of pollen per flower increase, the distance needed to forage decreases [118]. This is demonstrated by *A. mellifera*, which have been shown to alter their foraging distances throughout the year as the availability of flowers changes [119]. Although variations persist in the foraging range, the size of a bee is the best indicator for how far it will be capable of foraging. This is most important for small orchard bee species such as *Lasioglossum* and *Ceratina* spp., because the abundance of small bee species significantly decreases as the distance from nesting habitat increases [120]. In general, small bees need closer nesting habitats to accommodate their shorter foraging range. However, with increasing floral resources, more bees are needed to provide pollination coverage.

Information on specific species’ foraging distances is scarce, though the buff-tailed bumble bee (*B. terrestris*) has been studied by several researchers (Table 2)*. Bombus terrestris* workers measure around 20–23 mm in anterior–posterior length, and as documented in a mark-recapture study, the majority of workers were shown to forage within 100 m of their hive and had an average foraging distance of 267.2 m ± 180.3 [118]. Due to all the variables present, the maximum foraging distance is often reported, but inconsistencies exist. Mark–recapture studies have documented maximum *B. terrestris* foraging distances of 800 m [118], 1500 m [121], and 1750 m [122]. Interestingly, Carreck et al. (1999) [123] and Osborne et al. (2008) [121], using harmonic radar, recorded maximum *B. terrestris* foraging ranges of 550 m and 630 m, respectively. When the *B. terrestris* foraging distance was analyzed with molecular analysis, distances of 312.5 m [124] and 758 m [125] were reported. Male *B. terrestris*, which do not forage but can transfer pollen, have been documented flying a distance of 9900 m from their hive [126]. The variation in these documented flight patterns highlights the complexity of variables which determine how far a bee will fly within its lifetime.

For most species, there is a lack of information on foraging distance. Due to the variability previously discussed, comparing different species in different studies would not be appropriate; however, the following studies are mentioned for their relevance to orchard bees. The blue orchard bee, *O. lignaria*, has been documented as having a maximum range of 600 m [127]. Geib et al. (2015) estimated the average foraging range of four *Bombus* spp. (mean ± SE), reporting *B. balteatus* (85.4 m ± 15.0), *B. flavifrons* (23.8 m ± 10.1), *B. bifarius* (110.25 m ± 41.7), and *B. sylvicola* (74.7 m ± 56.3) [128]. *Andrena cineraria*, an average-sized bee measuring up to 15 mm in length, has been documented as having an average foraging range of 25 m² [76]. Although these data do not apply in different habitats or years, they provide insight into these complex interactions (Table 2).

**Table 2:** Foraging ranges reported for different bee species found in orchards. Maximum ranges found for each species and average ranges are reported for all species *. If only one value was found, the average is the same as the maximum.

Species	Max Reported Range (m)	Average Reported Range (m)	Bee Size (mm)	Number of Studies
*Andrena cineraria*	300	300	10–14	1
*Andrena vaga*	600	555	11–15	2
*Andrena barbilabris*	530	515	10–12	2
*Anthophora abrupta*	12,500	12,500	14–17	1
*Bombus balteatus*	220.5	85.4	11–14	1
*Bombus bifarius*	220.5	110.25	8–14	1
*Bombus flavifrons*	202.5	23.8	9–12	1
*Bombus sylvicola*	290.5	74.7	10–14	1
*Bombus terrestris*	2800	1137.6	20–23	8
*Bombus terrestris (male)*	9900	9900	20–23	1
*Hoplitis adunca*	1400	1400	8–12	1
*Hylaeus punctulatissimus*	1100	1100	6–8	1
*Osmia lignaria*	600	600	11–12	1

* Information based on Zurbuchen et al. [79] and Geib et al. [128].

### 3.4. Orchard Crop Preference (Food Sources for Adults and Their Offspring)

As discussed previously, bees can specialize or generalize their flower foraging for different flowers, and not every flower visit consists of gathering pollen. This is because adult bees usually do not require pollen as larvae do. Bees gather pollen to make provisions for their unhatched young. Each offspring is provided with a pollen loaf which is primarily pollen gathered by the mother bee [92]. The pollen is often mixed with nectar, but some bees (such as *Centris* spp.) will also mix oils into the pollen loaf [129]. With the exception of three *Trigona* spp. in Panama, pollen serves as the only nitrogen source in a bee’s diet, thus fulfilling the protein requirements for developing larvae [92]. Nectar is not only collected to mix with pollen, but is also the primary food source of adult bees. Some interesting behaviors have been documented in *O. cornuta*, in which some individuals will mix pollen from two to six different species to offset unfavorable metabolites in some of the pollen [130]. The quality and availability of these two resources within different flower species contribute to bee preferences for certain floral species.

Generalist bee floral preferences are not always well understood, because many different variables such as pollen quality, floral odors, flower shape and color, and competition with other pollinators can all impact preference. However, many generalist bees, such as *Osmia* spp., *Andrena* spp., and *Colletes* spp., show preferences for orchard flowers. *Osmia lignaria* strongly prefers Rosaceae crop species in orchard settings [99]. This could be due to flowering times, because many *Osmia* spp., *Andrena* spp., and *Colletes* spp. are active early in the year when orchards are blooming and other floral resources have not yet become available [131,132,133].

### 3.5. Time Matching with Flowering Period

Orchard tree species have narrow flowering periods of 2–4 weeks, but there is an even narrower period in which the majority of flowers are available for pollination, and it is important to have a species-rich and abundant bee community during that time to increase yields [134]. Orchard crops, such as apples, flower at different times each year in response to many environmental variables [135]. This response to the environment is different in plants compared with bees, potentially creating asynchrony with specialist obligates [136]. This makes early season generalist bees important for the pollination of early blooming orchard species. 

## 4. Diversity of Bees in Orchards

**Figure 3:**
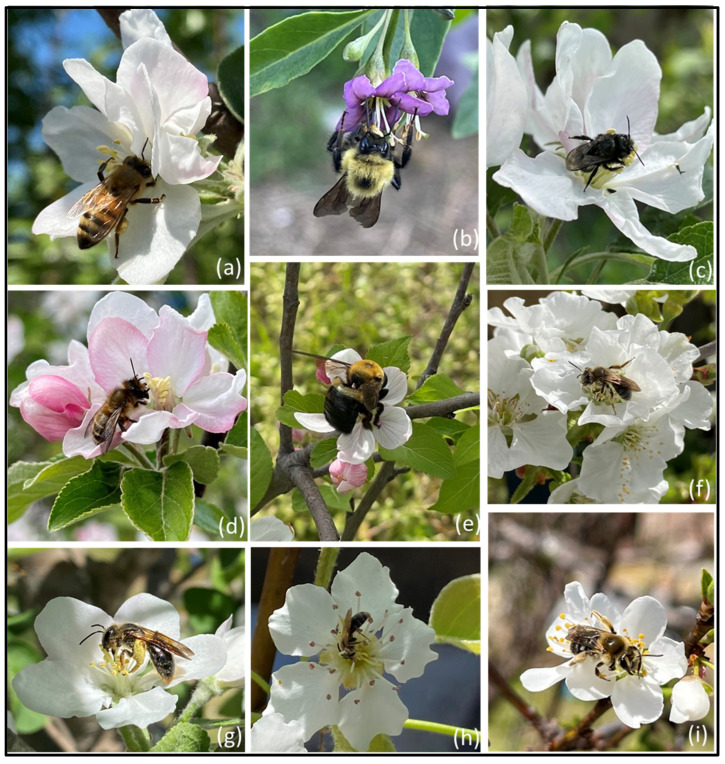
Different species of bees commonly found in tree fruit orchards in many fruit-growing regions in the United States. Cavity-nesting bees: European honey bee, *Apis mellifera* (**a**), bumble bee, *Bombus* sp. (**b**); tunnel-nesting bees: blue orchard bee, *Osmia lignaria* (**c**); Japanese orchard bee, *Osmia cornifrons* (**d**); Eastern carpenter bee, *Xylocopa virginica* (**e**); and ground-nesting bees: different mining bees (*Andrena* sp.) foraging on cherry (**f**), apple (**g**), pear (**h**), and plum (**i**) flowers. Pictures by N. Joshi.

Wild pollinators play a pivotal role in pollinating many agricultural crops (Figure 3) [137,138]. Historically, heavy reliance has been placed on *Apis mellifera*, the European honey bee. However, studies have shown that crop yield and size are positively impacted by wild bee visitation [137,139]. Therefore, having a wide range of bee diversity in orchards would be beneficial. Various studies have been performed to document bee diversity in orchards and show bee population variation based on the geographical location, degree of orchard management, and flora diversity within and surrounding the orchards [140,141,142]. For example, a study performed by Evans et al. (2021) on 18 macadamia orchards showed that cavity-nesting bees accounted for 60.5% of flower visitors. Stingless bees came in second, accounting for 35.8% of all flower visitors. Stingless bees in this study only visited orchards within 100 m of their nests; honey bees were not impacted by distance [143]. Bee species have varying flight ranges that they are willing to travel for resources, which could limit their ability to visit orchards. A similar study performed by Kammerer et al. (2016) surveyed bees in apple orchards and looked at the effect of plant diversity across multiple habitats on bee diversity [142]. The apple orchards studied were typical of those found in the Appalachian Mountain region—humid climate, well-drained soil, and a heterogenous landscape composed mainly of meadows and forests. After specimen collection, 118 bee species were identified: ground-nesting bees included Andrenidae, Halictidae, and Colletidae families, cavity-nesting bees included the Apidae family; tunnel-nesting bees included the Megachilidae family. The most prominent species were *A. mellifera*, *Augochlora pura*, *Ceratina calcarata*, *Lasioglossum pilosum*, and *Bombus* spp. Overall, 66% were ground-nesting, whereas tunnel- and cavity-nesting bees comprised 26% of the population. The remaining 9% were cleptoparasitic bees [142]. 

Another study involving apple orchards was performed by Sheffield et al. in 2013 [144] in Nova Scotia, Canada. This study focused on the diversity of the primarily tunnel-nesting family, Megachilidae, across abandoned, semi-managed, and managed apple orchards. Management included pesticide use, tilling, habitat destruction for crops, and general disturbances to the natural environment. Over the course of the study, 18 distinct species representing two genera were recorded. *Osmia tersula* was the most commonly trap-nested bee throughout all habitats. According to the study, the presence of bees within the set habitats was dependent on three factors: the number of suitable nesting sites, the amount of nest-building material, and the availability of food sources [145].

Further studies conducted on apple orchards reveal the potential diversity of bees across apple orchard ecosystems. Russo et al. (2017) observed the relationship between functional traits of various bee species across apple orchards in New York state versus seed set [146]. Between 2008 and 2013, they were able to detect over 100 bee species. The bees were categorized into seven classifications: bumble bees (cavity-nesting), honey bees (cavity-nesting), large *Andrena* (ground-nesting), small *Andrena* (ground-nesting), *Osmia* (tunnel-nesting), small black bees, and metallic green bees. Interestingly, even though many bee species were recorded, only a small number made up the vast majority of the abundance. Honey bees were the most common and equaled the abundance of wild bees altogether [146].

Bee diversity is also impacted by the crop species grown and the surrounding flora in an area. For example, a study by Tepidino et al. (2007) surveyed pear, sweet cherry, apricot, and apple orchards in the Capitol Reef National Park in Utah and found that significantly higher number of native bees in pear orchards versus apple orchards [147]. Moreover, honey bees were less common on sweet cherries than apricot. This could be due to pollen and nectar preferences, flower shape, proximity, or other factors. Overall, ground-nesting *Andrena prunorum*, *Anthophora porterae*, and *Lasioglossum pulveris* were common native bees and the native, tunnel-nesting *Osmia lignaria* species was a common sighting as well [147]. The tunnel-nesting *Megachile* spp., which are commonly used for alfalfa pollination, bumble bees, commonly used for tomato pollination, and *Nomia* and *Osmia* spp., commonly used in apple pollination, are all impacted by floral diversity [72,148]. These species utilize those plants for both nest construction and food. Another study, working strictly with tropical mango orchards, aimed to assess the relationship between local landscape and habitat characteristics and the diversity of visiting bees [139]. Within 24 mango orchards, the team trapped 3,842 individual bees from 28 species. Over 92% of the trapped bees were *Apis* spp. *Apis florea* comprised 82.9% of all bees alone. *Apis cerana* followed with 9.1%, then *Lasioglossum* spp. with 1.9% [139]. Bee diversity can be impacted by spatial and temporal factors [61,115,143], floral diversity presence [72,143], and agricultural management practices [140]. The most common solitary, ground-nesting bees are the *Andrena* spp., *Colletes* spp., *Lasioglossum* spp., *Halictus* spp., and *Augochlora* spp., which can comprise up to 66% of all native bees pollinating orchards [149]. Other bee species have been observed in orchards and add to their diversity, but only reflect a small fragment of their abundance. Bee diversity fluctuates from orchard to orchard; common sightings in one orchard may rarely occur in others nearby.

### Genetic Diversity of Orchard Bees

The genetic diversity of bees associated with fruit tree orchards can have important ecological consequences at the population, community, and ecosystem levels. Therefore, understanding the ecological implications of the genetic diversity of bees is critical to preserving native populations and improving fitness or pollination [150]. Genetic diversity refers to the origins and variation in traits in a particular group of organisms (e.g., populations or species) and their role in the evolution of sexual reproduction. The level and type of genetic variation can affect the rate of evolutionary change within populations [151]. Parameters measuring genetic variation within a population include allelic diversity, allelic richness (e.g., microsatellites and DNA sequences), heritability, genetic variance, and nucleotide diversity [152,153]. Several studies have examined the diversity of orchard bees, including species in the genera *Bombus*, *Andrena*, *Lasioglossum*, and *Osmia* [17,154,155,156]. In this section, we briefly summarize some aspects of allelic richness and the genetic diversity of these different groups of bees, particularly emphasizing the genus *Osmia* because of its critical role in tree fruit pollination.

*Bombus* spp.: bumble bees are distributed across many ecosystems [155]. Nearly 260 species have been described worldwide, with approximately 60 of them occurring in North America. Several studies have demonstrated changes in population genetics in bumble bee species [17,154,156]. For example, Cameron et al. (2011) identified changes in 8–11 microsatellite loci in *B. pensylvanicus*, *B. occidentalis*, *B. bimaculatus*, *B. impatiens*, *B. vosnesenskii*, and *B. bifarius* [17]. In addition, other studies documented that significant differences in allelic diversity among populations were closely associated with ecological niche [157] and environmental changes [154].

*Andrena* spp.: Approximately 1,560 species have been described, distributed throughout all continents except Australasia and Antarctica. In North America, 511 species have been reported [158]. Exeler et al. (2010) suggested a strong genetic exchange among populations of the genus, with some exceptions, such as *Andrena fuscipes* [159].

*Xylocopa* spp.: Approximately 500 species have been described, from which nine have been reported in North America [158]. Several studies have shown the impacts of human activities on carpenter bee gene flow [140]. Using 10 microsatellite loci analyses, Ballare and Jha (2021) found significant levels of high genetic relatedness in microhabitats, suggesting dispersal at the regional scale [160]. However, other studies have indicated high sensitivity to landscape changes that may limit gene flows as the landscape becomes more fragmented because of human activities [161,162,163,164].

*Osmia* spp.: This genus has approximately 353 species [81] that live in different ecosystems of North America, southern Europe, northern Africa, the Middle East, central Asia, and southern Africa [158]. In North America, 130 *Osmia* species have been reported, of which a large majority occur in the western United States. For example, 88 species have been reported in California [165], 76 in Colorado [166], and nearly 50 in Utah. In the southeastern United States, 55 species have been reported in Florida, and 14 species in Louisiana [158]. *Osmia* species diversity is consistently low in northern temperate regions of the United States, with only 18 species being reported [158].

Both native and introduced species contribute to pollinating fruit trees such as almonds, apples, and cherries [72]. Other species such as *O. ribifloris* and *O. aglaia* have been investigated as potential managed pollinators of blueberries and raspberries [167,168,169]. The reliance on *Osmia* spp. for crop pollination ranges from conservation management to the manipulation of nest structures. Conservation management involves changing farming practices, especially pest management practices, to be less threatening to pollinators. Integrated pest and pollinator management (IPPM) is designed to manage pests while preserving pollinator health [170]. *Osmia* spp. are cavity nesters; artificial nests can be made from plant stems, wooden blocks with drilled holes, and other devices that will attract female bees for nesting. These artificial nests can be situated in crops and refrigerated to adjust spring emergence times [171].

Genetic diversity can also change in response to environmental variation and constraints. Monitoring programs for assessing the abundance of two exotic and six native *Osmia* species over fifteen years in the Mid-Atlantic United States indicated that native species had experienced substantial declines during this period while exotic species fared much better, with the exotic *O. cornifrons* (introduced from Japan) remaining stable and *O. taurus* (introduced from China and Japan) increasing by 800% [172]. Other studies have shown that only the population of one species, *O. lignaria*, is increasing, while 39 species are remaining stable, and 17 species are declining [172]. Although it is difficult to ascertain the mechanisms that underlie the community structure, habitat changes, natural enemies, and interspecific competition might favor exotic species [173].

Another area of research inquiry is evaluating the genetic diversity of the *Osmia* genus in North America, which can give clues to the stressors that various *Osmia* species have encountered. Few studies have explored patterns of genetic diversity, and only two studies have investigated the genetic structure of *O. bicornis* and *O. cornifrons* [174,175,176]. For example, Beadle et al. (2019) used microsatellite analysis to investigate the genes associated with susceptibly and tolerance to environmental stressors, such as pollution [174]. Other studies have explored changes between females and males, which identified 34% of genes which were significantly upregulated in females relative to males in response to pesticide exposure. Splicing analysis showed that 8.64% of genes were significantly spliced between the sexes [175]. Changes in gene expression were associated with 107 physiological processes, including xenobiotic detoxification [175].

Functional genomics analyses can provide valuable information about genome-wide architecture and changes in gene expression in response to environmental stress or pathogenic infection. These studies may also provide valuable information regarding intrinsic differences in gene expression between different life cycle stages or sexes [177,178]. For example, differential gene expression has been identified across clades, which may mirror differences in biochemistry, physiological pathways, genomic architecture, and life histories [179]. However, more research is needed to better assess the impacts on the genetic integrity of native species and the implications for ecological interactions, foraging performance, and response to climate change.

## 5. Environmental Threats and Multiple Stressors Affecting Orchard Bee Communities

### 5.1. Pesticide Exposure and Hazard

Bees are exposed to many hazards in the field, which can negatively impact their foraging ability and pollination service. One such hazard in orchards and other agricultural landscapes is exposure to toxic pesticides, which can occur in several ways. Bees foraging in commercial crops, especially, can be caught in foliar spray applications of pesticides. They can be exposed to higher concentrations of pesticides in this way and can have more severe reactions. Honey bees (*A. mellifera*) and stingless bees (*Hypotrigona ruspolii*), for example, had higher mortality after direct spray exposure to the herbicide glyphosate than when they were exposed to dried pesticide residues [180]. Similarly, a bacterial toxin, spinosad, and two neonicotinoid insecticides, imidacloprid and clothianidin, were more toxic to *A. mellifera* after direct spraying than when bees were exposed to dried residues [181]. There is also risk, however, of environmental residues and the accumulation of pesticides near treated fields. The application of systemic insecticides can translocate through the plant tissue and be present in pollen and nectar [182,183]. In fruit orchards, these systemic pesticides are an integral part of fruit growers’ pest management practices [184,185,186] and are applied via foliar applications. Recent studies have identified a low amount of residues in the pollen and nectar of apple flowers [187]. Many systemic pesticides used in orchard crops (such as apples) are known to be toxic to honey bees as well as the solitary bee *O. cornifrons* [188,189]. Pesticide treatments to non-orchard crops can also contribute to residues in and around bee nests. One survey found clothianidin residues in maize and soybean pollen in both sprayed and unsprayed fields, although concentrations were much lower in the untreated fields [190]. Stored honey bee pollen can also contain pesticide residues at high concentrations. A honey bee hive near a treated maize field had pollen stores with up to 88 ppb clothianidin [190]. For ground-nesting bees, such as bumble bees (*Bombus* spp.), mining bees (*Andrena* spp.), and many sweat bees (Hymenoptera: Halictidae) [54,191], as well as mason bees (*Osmia* spp.), which use mud in their nest construction [70], exposure to pesticide residues in the soil is also a risk. The soil of crop fields treated with foliar sprays or planted with systemic pesticide-treated seeds can contain high levels of pesticides, especially directly following planting or spray application [190,192]. Finally, water sources can pose a pesticide exposure risk to bees. Many water sources near treated fields can accumulate pesticides [192,193], and in smaller bodies of water, such as puddles, concentrations can become very high, such as the 131 ppb imidacloprid found in one such water source in Maryland [3]. Bees, themselves, can also be found exhibiting pesticide residues. A survey of native North American bees in the western United States found that 70% had traces of at least one pesticide, while 48% had at least two [194].

After exposure to pesticides, bees can experience several negative effects to their health, foraging ability, and fecundity, depending on the type of pesticide and the concentration. Insecticides are generally the most directly toxic to bees. Insecticides impact the nervous system, development, respiratory system, or the midgut of both target insect pests and non-target beneficial insects, such as pollinators [195]. Many of the most commonly used insecticides, including pyrethroids, organophosphates, neonicotinoids, spinosyns, and sulfoximines, are neurotoxic, and can be both synthetic or natural, derived from plants or bacteria [196]. These neurotoxic insecticides can increase the mortality of several bee species [197,198,199], and negatively impact their fecundity and development [200,201,202], immune response [203,204,205], behavior [182,206], and foraging ability [199].

Herbicides and fungicides are often considered harmless to insect pollinators, though they can also negatively affect bee health and functioning [207]. Herbicides such as dicamba and glyphosate can reduce the availability of flowering plants for bee foraging [208,209]. Herbicides and fungicides can also alter the gut microbiome communities of bees [210,211]. Social corbiculate bees, such as *Apis* spp. and *Bombus* spp., have consistent gut symbiont communities, which can improve the bees’ pathogen resistance [212,213], aid in detoxification [214], and play a role in growth and development [215]. By altering the community structure of the bee gut symbionts, herbicide and fungicide exposure could also affect the health benefits provided by these microbial symbionts [216,217]. For solitary bees, gut microbiome communities tend to be more varied and more influenced by the environment [218,219]. Little research has been conducted into the impact of pesticides on the gut communities of solitary bee species. Continued research into the impacts of pesticides on different bee species can help to better protect valuable insect pollinators from the adverse effects of pesticide exposures. Developing pest management strategies using an integrated pest and pollinator management (IPPM) framework is important in tree fruit crops [185].

### 5.2. Arthropod Pests and Other Natural Enemies

Bee pollinators can also be preyed upon and impacted by a variety of predators, parasitoids, and cleptoparasites. With the introduction of the giant hornet (*Vespa mandarinia*) to North America, there have been concerns about its impact on honey bees, bumble bees, and other Hymenopteran pollinators [220,221]. *Vespa mandarinia* workers will kill individual bees outside of their nests and attack social bee colonies en masse, killing the adult bees and taking the bee brood to feed the hornet larvae, a hunting strategy that can wipe out bee hives [222]. Other bee predators include bee wolves (*Philanthus* spp.). One study found that a single bumble bee wolf (*P. bicinctus*) could reduce bumble bee population density and foraging activity within its hunting range [208]. Crab spiders (Family: Thomisidae) often hunt near pollinators’ preferred flowers. Their hunting activity can alter bumble bee foraging behavior, with bees choosing safer, but less desirable flowers over those with a risk of spiders [223].

Parasites and parasitoids may target the bees, themselves. *Monodontomerus* spp. wasp larvae are oviposited into the prepupae and pupae of Megachilid bees, including *Osmia* spp. and *Megachile* spp., and then consume and kill their hosts [132,224]. Twisted-wing insects (Order: Strepsiptera) are endoparasites of other insects, including many Hymenopterans [225]. One Strepsipteran species, *Stylops advarians*, is a parasite of the solitary *Andrena milwaukeensis* bee, and alters the alimentary canal and inhibits ovarian development in its host [226].

Several cleptoparasitic species live within the nests of bees, often feeding on the pollen and nectar gathered for the bee offspring. One group of these are the cuckoo bees, which includes several genera: *Coelioxys*, *Nomada, Sphecodes*, and *Stelis* [227]. These bees do not collect pollen, but instead lay their eggs in the nests of other bees, leaving their offspring to eat the host’s food stores [228]. *Chaetodactylus* spp. mites will feed on the pollen provisions of *Osmia* spp. bees, and in doing so, can reduce the amount of food available for the bee larvae [40,132]. Other cleptoparasites such as chrysidid wasps, blister beetles, and velvet ants can also enter bee nests [229,230]. Some opportunistic nest parasites can also be present, such as booklice [231] and dermestid beetles [232].

### 5.3. Parasites and Diseases

Many parasites and pathogens, including various viruses, bacteria, fungi, and protozoan parasites, are known to infect bees. Of these, most have been studied in *Apis* spp. and *Bombus* spp. bees, although some are known to infect and cause lethal and sublethal effects in other bees as well [141].

Viral prevalence amongst wild and managed bees can be high, with one survey finding that over 80% of wild bees, including members of the families Apidae, Megachilidae, Andrenidae, and Halictidae, had at least one pathogenic virus present [233]. The pathogenic effects of these viruses, such as deformed wing virus (DWV) and black queen cell virus (BQCV), are best known in honey bees. However, they are able to replicate in several solitary bee and bumble bee species [234]. In the case of DWV, the infection can cause wing and leg deformation, discoloration, and bloated abdomen in honey bees and bumble bees, but its pathogenicity in solitary bees is less studied [235]. With such viruses, which have a wide range of potential hosts, there is also concern for spread between wild and managed bee populations [233]. 

Several bacterial pathogens can also cause negative health effects in both *Apis* and non-*Apis* bees. *Paenibacillus larvae* is the cause of American foulbrood, a devastating disease of honey bee larvae [235], although another *Paenibacillus* sp. has also been correlated with higher larval death in the solitary mason bee *Osmia bicornis* [236].

Fungal pathogens, such as *Ascosphaera* spp., the cause of chalkbrood, can infect the larvae of both honey bees and certain solitary species, including *Megachile rotundata*, causing increased larval mortality [141,237]. *Nosema* spp. are known to infect social bees. *Nosema bombi* causes a systemic infection in *Bombus* spp. bees, which can decrease worker and male bee survival and cause infected males to be less fertile [238]. *Nosema ceranae* was originally a fungal pathogen of *Apis* spp., but has jumped host species to infect bumble bees as well [239]. 

Bumble bees can also be infected by protozoan parasites, including the trypanosome, *Crithidia bombi* [213], and the alveolate, *Apicystis bombi* [240]. The importation of non-native bumble bee species, such as *Bombus terrestris*, can introduce parasites, such as *C. bombi*, to novel bee populations, including the vulnerable South American species *Bombus dahlbomii* [241]. More research is still needed into the pathogens of bees and how they spread in order to better quarantine and protect pollinator populations from disease.

### 5.4. Lack of Nesting Habitats

The loss of natural habitats has contributed to inadequate nesting sites for bees in many areas. As has been discussed previously, wild bees have differing nesting preferences, with some bees digging into soil, inhabiting abandoned burrows, nesting in found tubes, or building their own tunnels in wood. There are also species-specific preferences within these broad groups. Within the ground-nesting bees, for example, preferences can vary greatly as far as soil moisture content and texture [191]. For tunnel-nesting bees, different materials can be used for nest construction, such as mud, leaves, and pebbles [67,70]. Natural and semi-natural habitats can provide a wide variety of substrates and building materials for bee nests, and can thus support a higher diversity of bees than unbroken monoculture landscapes and urban areas [242].

Habitat loss is a major cause of the decline in orchard bee communities; however, it is hard to specify population decline due to habitat loss because of two major reasons: (1) bees being dependent on a variety of nesting substrates; and (2) the diversity of bees for nesting preference [59]. In general, the availability of pollinator habitats, nesting substrates, and nest building materials helps in the establishment of bee communities [243]. The accessibility of bare ground, nesting cavities, steep and sloping ground, abundant flowering species with pithy stems, and the occurrence of pre-existing cavities or burrows are conducive for pollinators to make nests [244]. Bees use hollow plant stems, cardboard papers, or wooden laminar nests to develop trap nests [57]. Nesting and foraging resources are necessary for structuring bee communities in an ecosystem [244]. Ground-nesting bees prefer areas with well-drained soils [244]. Shrub and woodland habitats with a range of post-burn age (intermediate age sites), flower richness, and moderate grazing intensities increase the abundance of bees [245]. Developing more landscape heterogeneity and boosting the quality of semi-natural habitat can help enhance and conserve pollinator diversity by providing more resources for those pollinators [246]. 

The availability of ancillary nesting materials and season-long floral resources along hedgerows around orchard are necessary for the sustainability of bees in fruit orchards [247]. Arranging nest boxes more densely around orchards with a moderate number of nest tubes per box increases the numbers of native and honey bees in fruit orchards, leading to increased fruit setting, improving overall farm profit [248]. High nest densities increase bee populations, as bees in orchards with inadequate nesting sites are more likely to leave the orchard and seek nesting sites in other areas [99]. Bee hotels and nest boxes have been reviewed for their bee occupancy rate. The average occupancy rate for bee hotels was 37.1%, whereas that of nest boxes was 13.8% [249]. The sizes of bee hotels and next boxes, and the place of installation, did not affect the occupancy rate [249]. Prior studies have recommended bee hotels as more favorable by solitary bees, whereas social bees such as bumble bees and honey bees prefer nest boxes [250]. An approximately 20–50% increase in bee habitat diversity within a 1 km radius increases the setting of fruits by up to 150% [251]. Increases in wild bee visitation and the setting of fruit were linearly correlated with an increase in wild bee habitat in fruit orchard [251]. In summary, an increase in nesting density with wildflower planting fulfills the critical resources for bee conservation in orchard agroecosystems [252,253,254].

### 5.5. Lack of Diverse Floral Food Resources

In addition to the loss of nesting habitats, converting natural and semi-natural lands into intensive agricultural or urban landscapes can reduce the availability of food resources for bees. There are several causes of this habitat loss, including agricultural expansion, urban development, and desertification from improper management [242,255,256]. Monoculture crop systems, even monocultures of bee-attractive flowers, can alter bee health and the population dynamics of pollinators [257,258]. Both the amount and the diversity of pollen available to bees affects their nutrition. In the case of many generalist feeders, having a variety of pollen types in an area allows them to mix pollen and collect higher quality pollen. *Osmia lignaria*, for example, a generalist mason bee, chose to mix pollen even when they had to travel longer flight distances to do so [259]. For generalist honey bees, having access to a diversity of pollen (polyfloral resources) and higher protein pollen improved their immunocompetence [256,260], and in bumble bees (*B. terrestris*), low-protein diets lowered their resistance to *C. bombi* parasites [261]. Specialist feeders are less successful in areas that do not have their preferred flower resources, and can have more trouble adapting to habitat conversion than generalists [242,262]. For North American bumble bees, species with narrower diets were more likely to experience population and range declines [263], which could be due to a lack of access to their preferred diet.

Losses of natural habitat, and therefore loss of more diverse floral resources and nesting sites, have correlated with declines in bee pollinator abundance and diversity. In Costa Rica, greater urbanization was associated with bee declines [264] and visitation rates of pollinators [265]. In Pennsylvania, apple orchards that did not have nearby natural habitats had lower visitation rates of *A. mellifera*, *Bombus* spp., and solitary orchard bee species compared with orchards that had forest and other natural patches within 250 m [137]. Providing more diverse floral plantings and preserving natural habitats near bee-pollinated croplands may help to preserve populations and pollination services of bees.

## 6. Management and Conservation of Orchard Bees

Although many stressors can impact wild bee populations and reduce their pollination efficiency, many strategies can be implemented to mitigate these risks [266]. Additionally, managed non-*Apis* bees can require different types of nests and be active at different times of the year when compared with honey bees. Understanding the unique diets, nesting habitats, and activity periods of these bees can help orchard producers keep non-*Apis*, alongside or independently of honey bees, within orchards.

### 6.1. Strategies for Enhancing Orchard Bee Nesting Habitats

Orchard bees have limited flight ranges from their nesting locations and frequently return to their nests to stock pollen and other floral products [267]. Therefore, it is crucial to conserve and/or manage nesting habitats near the target crop and other floral resources that provide food throughout the foraging season. Although most bee species are generalists, many species are specialists, with limited floral preferences and nutrition requirements, and will choose nesting sites near their host plants [268,269,270,271,272,273]. Therefore, a diversity of native plant species near nesting sites will attract the highest diversity of orchard bee species. The closer pollen and nectar resources are to bee nesting sites, the less energy they must spend on commuting. This results in higher food provisioning and more offspring [92].

#### 6.1.1. Ground-Nesting Orchard Bees

Most wild bee species nest in the soil. Estimates range from 64% [274] to 83% of all bee species [275]. Nesting underground is thought to be ancestral among bee taxa [276]. Ground nesters either excavate or re-purpose burrows for the protection of eggs, larvae, and pupae. The soils in orchards are often too compacted by mowers and other equipment to be suitable for ground-nesting bees. Therefore, setting aside open areas, which do not have vehicle traffic and where plant cover is kept to a minimum, is an important management strategy. Developing progeny may spend several months underground, with some requiring more than a year [277,278].Thus, protection from tilling and vehicle traffic is crucial. If mowing is necessary, it should be carried out in the late fall or winter, after the bumble bee colonies have died for the year and the queens are dormant [279].

Different species require different moisture contents and have varied strategies to manage extremes of dry and saturated conditions; therefore, soils should generally be well-drained to prevent flooding, while providing the moisture required for larval development. Most ground-nesting bees and other ground-nesting Hymenoptera utilize sandy soils that are easier to excavate and provide better drainage [191,280]. Cane (1991) found that among 32 species of North American ground-nesting bees, nests contained sand percentages in soil that ranged from 34% to 94% [191]. Sandy soil, however, can be deficient in the clay content needed for tunnel stability [281]. Some species prefer nesting sites with sizable rocks that may act as visual cues to help the bees locate their nest quickly after returning from frequent foraging trips [282]. Stones absorb solar radiation and retain heat, as well, helping to regulate underground temperatures. Warmer soils allow the bees to have earlier foraging start times each day [283]. South-facing slopes with morning sun are ideal. Different slopes, from vertical to flat, will attract different bee species and can provide protection from weather [279].

Although ground-nesting bee species are more common, they have not been studied as thoroughly as cavity and tunnel nesters; therefore, specifics on the nesting biology of most species are still lacking in the literature. More specifics on what is known about the ground-nesting habitat of various bee species can be found in a 2020 review by Antoine and Forrest [284].

#### 6.1.2. Cavity and Tunnel-Nesting Orchard Bees

Some of the more productive bee species, in terms of pollination, nest in rotting logs or the woody stems of shrubs. Therefore, the proximity of sturdy-stemmed forbs, shrubs, and standing and downed trees to the target crop will provide more suitable nesting sites and increase pollination services by wild bees. A higher abundance and diversity of cavity-nesting bee species was found in orchards with a high species richness of flowering plants [285]. Some species have specific plant host preferences, so a diversity of native plants will attract the highest diversity of orchard bee species. Solitary wood-nesting bees comprise nearly 30% of the native bees in North America [279]. They will either tunnel into soft pithy centers of twigs, such as box elder, elderberry, or various cane berries; borrow into wood, in the case of carpenter bees, or find a cavity made by another animal, such as a wood-boring beetle larvae. A small but important group of bees tunnel into soft, above-ground rotting logs and stumps. Dead trees should be left in place or put into a pile, but not removed altogether because they provide critical nesting substrate. Tunnel-nesting bees also need various materials to construct their brood cells and seal their nests. Bees that do not secrete their own cellophane-like structure to line brood cells gather pieces of leaves, petals, floral oils, mud, fine pebbles, or tree resins [65,268,286]. Therefore, it is important to provide diverse native plants and protected areas with damp clay. 

Orchard management is relatively intense and may create unsuitable conditions for stem and cavity-nesting bees [144,257,287,288]. Vegetation adjacent to and between tree rows in the orchards is regularly mowed and herbicides are applied beneath trees to reduce competition for water and nutrients. This severely limits nesting options for tunnel-nesting bees. Rather than mowing, leaving hearty stems throughout the winter can be key to providing nesting habitat for orchard bees.

### 6.2. Proximity of Forest Habitat Improves Nesting Conditions for Orchard Bees

A survey of the wild bee community in an apple-growing region in Wisconsin showed that a high proportion of forest area in the surrounding landscape was a significantly positive predictor of wild bee species diversity in orchards [289]. A survey of three plant communities in and around apple orchards of Adam’s County, Pennsylvania, in the orchard, forest, and forest edge, showed that the forest edge provides the highest level of plant species richness. In one study, researchers found that orchards located within a landscape with more forest and species-rich forest edge had higher species richness of bees. The closer the forest edge was to the orchard, the higher the bee species richness and abundance [149]. Adjacent semi-natural habitats attract higher colonization rates and numbers of cavity-nesting bees (and beneficial predator wasps) and brood cells compared with apple orchards [285]. Patches of diverse native plant habitats intermixed within the agricultural landscape generally enhance the diversity and abundance within orchards [144,287,290,291,292].

Patches of forest habitat can also help provide ground-nesting opportunities. Forests provide a range of soil types and other substrates for overwintering, particularly for species that prefer to nest in or among leaf litter or vegetation. Soils in deciduous forests provide the additional resource of more organic matter in the form of leaf litter that would provide a softer composition for burrowing. Tunnels can sometimes be as deep as 36 inches below the surface [191]. Higher moisture contents of shady soils compared with exposed soils will also aid in a small insect’s ability to burrow. Forests provide tree resins used by some species to waterproof their brood cells.

Bumblebee queens often prefer north-facing protected sites where they can burrow to overwinter, often in soft hummus and leaf litter. These larger species of bees construct nests in small cavities, often in old rodent burrows, either underground or beneath fallen plant matter, or occasionally above ground in abandoned bird nests. Forests provide an abundance of these nesting options. Bumble bees prefer forest edge habitats where rodents are more common.

Trees and understory shrubs also provide abundant pollen and nectar resources and non-floral resources such as honeydew and sap that can provide food in times of scarce flowering resources [293]. Compared with agricultural settings, forests provide more hiding places from predators and parasitoids that target bees. Forests also provide cover during the intensive application of pesticides in agroecosystems which has been significantly linked to declines in the bee community. Most bee species are very sensitive to cold, wet, and windy weather; therefore, the proximity of forest habitat provides an important sanctuary compared with the exposure experienced in orchards and row crop fields. Forests also provide more protection in the case of severe weather. This will likely be more important as bees adapt to climate change.

### 6.3. Building Artificial Nests for Orchard Bees

**Figure 4:**
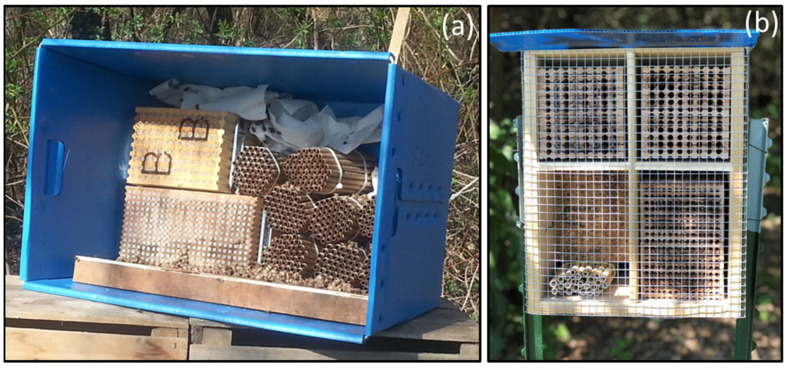
Nest substrates deployment (nesting tubes and wood blocks (**a**) and a nest box style bee hotel (**b**)) for tunnel-nesting bees found in orchards. Pictures by N. Joshi.

Higher tree fruit production profits have been seen with the use of artificial nest boxes [248]. Different nest substrates could be used for tunnel-nesting orchard bees (Figure 4). Tunnel nesters will use a variety of structures that mimic beetle holes in wood or hollow pithy stems. Simply drilling holes of different diameters into blocks of wood or tying bundles of straws or bamboo together can provide artificial nesting opportunities when natural options are limited or take time to establish. Nests should be erected at least four feet above the ground to raise them above the cool moist air that pools up at night. They should be mounted with tunnels positioned horizontally in a south-facing location which receives morning sun, but with protection from rain and extreme mid-day sun. A constructed roof over the block (as shown in Figure 4) can serve this purpose if shade trees are absent. The female bee always finishes the nest with male brood cells; therefore, holes must be deep enough to provide space for female brood cells [279]. Not only do females ensure future generations, but they are also the primary pollinators because they provision their nests with pollen and nectar. Cleaning out the holes each year will reduce infestations of parasites, fungi, and diseases. One advantage of using paper straw liners is that they are easy to clean.

Drilling holes into dry logs and placing them upright, similarly to a fence post, can simulate a beetle tunneled snag. Varied hole diameters will attract different-sized bee species. The tunnel entrances should be facing southward. To avoid flooding, tunnels should be drilled at a slight angle, so that water can drip out. Drill bits should be sharp and used at high speeds for smoother-grained interiors. Drilling with the wood grain, rather than against it, is also recommended for smoother tunnels [68]. Paraffin-coated paper straws can also be used to line the holes, although the range of diameters is small. If the tips of the straws are painted black or red, this can help to attract bees [279]. Some commercially available straws are semi-translucent for the easy inspection of nest contents.

Bumble bees have been known to occupy artificial structures as well. Small boxes or other containers such as empty flowerpots stuffed lightly with an insulating material, such as upholstery cotton, can sometimes effectively appeal to bumble bees. Hay bales are sometimes used as nest sites for bumble bees [294,295].

Among the provisions not mentioned that will encourage more nesting bees is access to surface water for hydration. Bees will not likely choose a nesting location too far from a source of drinking water. Bee baths or other structures should be provided if surface water is not naturally available.

More research is needed for precise artificial nesting strategies for most species of orchard bees. Overall, restoring diverse natural habitat types is the best course of action. The better the nesting options are for a wide variety of bee species, the stronger the pollinator community will be.

### 6.4. Establishment of Native Floral Plantings to Support Orchard Bee Communities

**Figure 5:**
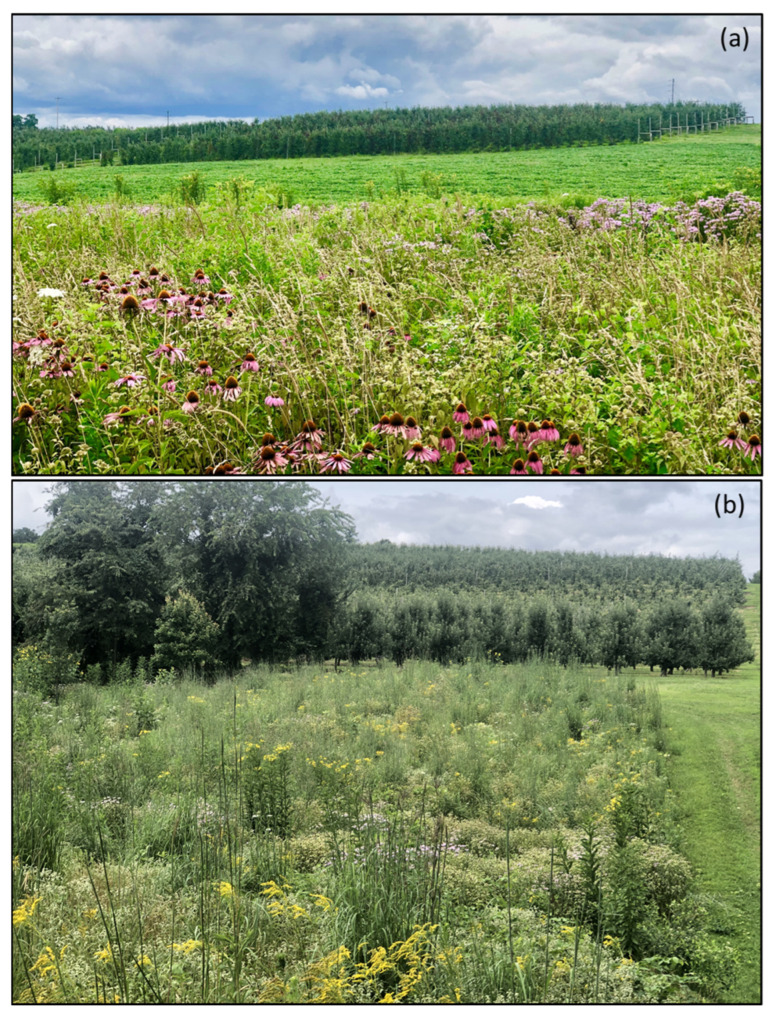
Examples of a native floral resource habitat establishment adjacent to apple orchards with high density (**a**) and traditional (**b**) plantings. Pictures by N. Joshi.

In light of pollinator declines, conserving habitat and supporting populations of orchard bees is an increasing concern among growers of pollinator-dependent fruit crops. In many high-value crops, growers have shifted from renting bee hives to relying exclusively on diverse wild bee species [296]. Diverse bee communities result in higher pollination rates compared to on the pollination done by only one or even a small number of bee species. Bee species vary in their pollination efficiency because they vary in their pollen transporting morphology [56,297,298], pollen load capacity [299], foraging distance from nesting site [117], duration of foraging trips and longer activity periods [300], and resilience in less favorable weather conditions [301,302,303], floral resource gathering strategy, such as prioritizing the collection of pollen over nectar [304], visiting male flowers more than female flowers [305], buzz sonication [306], and amount of non-foraging work (e.g. nest construction) performed [307]. Therefore, the conservation and management of bee habitat by establishing native floral resource plantings that support diverse bee species in commercial fruit farms is crucial for sustainable production (Figure 5).

The conservation of agricultural land with native flowering plants and grasses provides nesting habitat and pollen for pollinators [308]. Flowering plants provide pollen, nectar, and nesting sites for pollinators, which is necessary for the normal survival and reproduction of pollinators, and for development during different stages of life cycle [178,246,309]. Pollinators such as bees require diverse food resources during their developmental stages to fulfill their nutritional needs [310]. Nectar from flowering plants is the greatest resource for the reproduction and survival of insect pollinators [71]. The conservation of insect pollinators depends on the diversity of flowering plants [178,309]. Most bees need multiple forage species that persist during entire foraging season for bees from early spring to late fall [15]. Perennial plants in crop margins, which bloom at different times during spring, summer, and fall, can supply floral resources to pollinators throughout the year, thus supporting pollinators [311]. Flower-rich habitats not only attract bees, butterflies, and other pollinators [312], but also increases the abundance of pollinators and diversity [311]. The inclusion of specific plant species for foraging resources provides a strong positive effect on the floral-unit abundance of specific bee species [262]. In general, the enhancement of flowering plant species increases bees and other insect pollinators in apple, pear, and other fruit orchards [313].

Pollination dependence on only one bee species, such as honey bees (*A. mellifera* ), for crop production is risky, because a single species is more vulnerable to exposure to pesticides, pathogens, parasites, and habitat loss, compared to a robust, multi-species community [22]. Wildflower establishments along orchard edges promote wild and blue orchard bees (*Osmia lignaria*), which can minimize pressure on honey bees to meet pollination demands [314]. The establishment of wildflowers in the proximity of apple not only increases overall pollination services, but also positively affects size of apple and increases economic value [315].

Flowers strips increase bee/non-bee pollinator abundance during blooming season in apple orchards; such effects are more prominent in areas bordered with flower strips [316]. The number of bee visitations in avocado orchards was sixfold greater when native flower strips were planted close to avocado orchard compared with native flowering strips far from avocado orchard [317]. Visitation rates also differed based on flowering species with different corolla lengths in the flower and season of the year [317]. Multiple studies have concluded that most benefits for pollination are obtained from the visit of first 6–8 bees/1000 flower and decreasing thereafter [318]. Additionally, for optimal bee visits, 65–75% of female flowers are desired in the orchard, and considerations should be made regarding bee density because it significantly affects fruit production [318]. The enhancement of floral resources surrounding agricultural crops in order to promote the health of honey bees and other pollinators has been encouraged by the U.S. Department of Agriculture (The White House, Washington, DC, USA, 2014). Fruits such as apples are pollinator-dependent crops and pollination is crucial, specifically in the first week after bloom. The establishment of mixtures of native perennial flowering plant that can bloom during different time periods could provide season-long floral resources to bees that, in turn, benefit apple orchards for pollination [131,319].

Studies conducted in Australia and South Africa have concluded that the enhancement of flowering plants increases the functional richness of wild bees in both organic and conventional vineyards [319]. Open grassy areas around the apple orchard are better predictors for wild bees visiting apple orchards than ‘natural vegetation’ or an ‘open grassy area plus natural vegetation’ [320]. There was greater bee nesting at farms with wildflower planting compared with farms without planting [254]. The preference for nesting bees was predominantly for few subsets of available flowers (*Centaurea maculosa* and *Rudbeckia*) rather than most mixed wildflower plants [254]. In cherry orchards, landscape management with a semi-natural habitat and increased native flowering plants increases wild pollinator diversity, which results in the support setting of cherry fruit [321]. Bee abundance declined with the increased isolation of apple orchards from natural shrubland, negatively related to forest plantation; however, the presence of flowering ground cover greatly maintained bee assemblage in apple orchard [322].

Field-based studies have aimed to assess the effectiveness of restoring native plant communities to attract and retain a diversity of wild bee species. Some studies have also sought to determine optimal restoration strategies, such as higher initial establishment rates of sowing flower strips in the fall over spring, on former arable fields over former grasslands due to less competition from plants in the seed bank, using mixes of regionally sourced native seed adapted to the local biotic and abiotic conditions over mixes of cultivars [323] and mulching twice a year in March and August in arid environments [324]. Although plants with a high nectar production have been shown to be more important than plant diversity among plants that all bloom at the same time in small-scale resource plots [325], floral provisioning should include enough plant species diversity to provide the continuous blooming of high-nectar-producing plants throughout the pollinator foraging season, as well as diverse nesting site options [131] to attract more pollinator diversity and abundance throughout the year. Some plant species may have high pollen and nectar rewards, but low rates of establishment, and are therefore not ideal candidates unless follow-up re-seeding is practical [326]. Different bees have co-evolved compatible morphological features with different flowers; thus, bees differ in their preferred corolla widths and lengths [327,328], petal colors and patterns, nutritional provisions, bloom time, and flower density and textures. Therefore, the greater the diversity of flowers, the greater the diversity of bees. Research supports the hypothesis that the closer the floral resource is to the crop, the higher the bee visitation rates [329]. There is no clear choice between establishing adjacent plots of native plants versus planting hedgerows [330,331,332] or strips between crop rows, because specific farmscape designs for provisioning vary by crop system and geographical features. The suggested ratio of cropland to natural habitat varies among farming regions, but 25–30% is the average recommendation [279], with some studies showing that at little as 2–3% can be effective for some crops [309]. A review of field studies assessing the effectiveness of pollinator habitat provisioning shows that, in most agroecosystems, although more research is needed, the overwhelming consensus is that providing floral resources increases crop yields and profits.

More research is needed on specific bee species’ floral and nesting requirements. Some species are generalists, whereas some form close associations with specific flowering plant species. In the case of highly specific pollinator–plant relationships, the conservation of both species is often necessary if both are to persist into the future. More often, however, bees require a variety of plant species to provide all of their nutrition needs. High-density plantings provide refuge from extreme weather and pesticides, while attracting beneficial arthropods such as native predators and parasitoids [333], which contribute to the biological control of pest species, reducing the need for pesticide applications [334]. With the optimization of provisioned floral resources, farmers may increase yields and lower the production cost for many crops, while helping to slow down the loss of biodiversity worldwide.

### 6.5. Protecting Orchard Bees from Pesticide Hazards

Protecting orchard bee pollinators from pesticides’ negative effects requires assessing pesticide risks. The contemporary testing of pesticides tends to be short-term and focused on acute contact exposure [335]. Mortality is often the only endpoint measured, although pesticides can have severe sublethal effects, such as rigid and irreversible paralysis [336]. Honey bees are often used as surrogates for all other pollinator species [337], although different species of bees can have different sensitivities to pesticides [198]. Additionally, there is little monitoring and regulation following the approval of a pesticide [335]. Although it is necessary to use surrogates, because testing every bee species would be impossible, expanding the pollinator surrogates to include a bumble bee species or a solitary species could help improve risk assessments. More chronic and sublethal effects testing could also provide a more complete idea of the potential harm of pesticides to pollinators [337].

The type of pesticides and pest management strategies, as well as the timing of pesticide applications, can also influence which insects are exposed and how their communities are affected by exposure to pesticides [338]. Bees are diurnal foragers and most active in crop fields during bloom; thus, daytime foliar sprays pose the most risk to them. Although insecticides are often the most directly toxic to bees, recognizing the risks of herbicides and fungicides to bee gut microbiota, floral availability, and bee health is also important for protecting pollinators.

Although pesticide residues can be present in many environments, providing unsprayed floral resources, such as wildflower patches and hedgerows, can help bees to diet mix with pollen from untreated flowers and reduce their exposure to higher concentrations of pesticides and other toxins [130].

## 7. Conclusions and Recommendations

Fruit and tree nut cropping systems greatly benefit from the pollination services of bees. Managed honey bees have long been used for this service, but the supplementation of orchards with non-*Apis* species can improve yields and benefit many local wild ecosystems [4,28]. Bees across many families can be found in orchards, and many of these species, especially the solitary bees, are rarely studied or surveyed. These bee species can face multiple environmental stressors, from pesticide exposure, pathogen and parasite spread, and the loss of floral resources and nesting sites [141]. Although non-*Apis* bees can require different management and conservation measures from honey bees, many mitigation strategies can support honey bees, other managed bees, and a high diversity of wild bees [265,339,340,341]. Growing evidence supports the importance of conserving habitat with diverse floral and nesting resources within the proximity of apple orchards and other insect-pollinated crops to support a diverse assemblage of wild pollinators and provide optimal pollination services [131,259,298,339]. Reducing pesticide use and providing unsprayed floral plantings can also help protect bees from exposure to toxins. More research is needed to understand the specific nesting requirements of more bee species and how to best manage farming landscapes to provide for their needs. These mitigation measures are important, however, for protecting robust pollinator communities and maintaining their pollination services for orchard crop production.

## Data Availability

Data are available upon request.
